# Bioinspired Preservation of Natural Killer Cells for Cancer Immunotherapy

**DOI:** 10.1002/advs.201802045

**Published:** 2019-01-27

**Authors:** Rami El Assal, Lotfi Abou‐Elkacem, Alessandro Tocchio, Shannon Pasley, Sandro Matosevic, David L. Kaplan, Claudia Zylberberg, Utkan Demirci

**Affiliations:** ^1^ Bio‐Acoustic‐MEMS in Medicine (BAMM) Laboratories Canary Center at Stanford for Cancer Early Detection Department of Radiology Stanford University School of Medicine Palo Alto CA 94304 USA; ^2^ Molecular Imaging Program at Stanford (MIPS) Department of Radiology Stanford University School of Medicine Palo Alto CA 94304 USA; ^3^ Akron Biotechnology, LLC Boca Raton FL 33487 USA; ^4^ Department of Industrial and Physical Pharmacy College of Pharmacy Purdue University West Lafayette IN 47907 USA; ^5^ Department of Biomedical Engineering Tufts University School of Engineering Medford MA 02155 USA; ^6^ Department of Electrical Engineering (by courtesy) Stanford University School of Engineering Palo Alto CA 94304 USA

**Keywords:** bioinspired materials, biopreservation, cryoprotectants, immunotherapy, natural killer cells

## Abstract

The ability to cryopreserve natural killer (NK) cells has a significant potential in modern cancer immunotherapy. Current cryopreservation protocols cause deterioration in NK cell viability and functionality. This work reports the preservation of human cytokine‐activated NK cell viability and function following cryopreservation using a cocktail of biocompatible bioinspired cryoprotectants (i.e., dextran and carboxylated ε‐poly‐L‐lysine). Results demonstrate that the recovered NK cells after cryopreservation and rewarming maintain their viability immediately after thawing at a comparable level to control (dimethyl sulfoxide‐based cryopreservation). Although, their viability drops in the first day in culture compared to controls, the cells grow back to a comparable level to controls after 1 week in culture. In addition, the anti‐tumor functional activity of recovered NK cells demonstrates higher cytotoxic potency against leukemia cells compared to control. This approach presents a new direction for NK cell preservation, focusing on function and potentially enabling storage and distribution for cancer immunotherapy.

Natural killer (NK) cells represent our body's first line of defense against cancers.^1^, ^2^, ^3^ These cells play a key role in innate immune system, and have a unique ability to fight cancer cells without prior sensitization (unlike T and B‐ lymphocytes).^4^, ^5^ Recently, various cell lines (e.g., NK‐92, NK‐YS, and KHYG‐1) have been established with immunotherapeutic capabilities and potential to develop third‐party good manufacturing practice‐compliant cell banks (e.g., NantKwest, Culver City, CA, USA).^6^, ^7^ Although these cell lines are in clinical trials for treatment of various cancers (e.g., leukemia, lymphoma, and melanoma),^6^, ^8^, ^9^, ^10^, ^11^, ^12^ long‐term storage that facilitates off‐the‐shelf logistic distribution and repeated clinical administrations presents a challenge.^13^

Cryopreservation has emerged as a powerful tool to extend the shelf life of cells by maintaining them at sub‐zero temperatures and slowing down their metabolic activity.^14^, ^15^ During cryopreservation, cryoprotectants (also known as cryoprotective agents (CPAs)) are used to protect the cells from injury, which might result from the cooling and rewarming processes.^16^, ^17^ NK cells are conventionally cryopreserved using dimethyl sulfoxide (DMSO), as a cryoprotectant, in a slow freezing methodology.^18^, ^19^, ^20^, ^21^, ^22^, ^23^, ^24^, ^25^ A detailed description of existing cryoprotectants and cryopreservation methods is shown in Supplementary Table S1 (Supporting Information). Although cryopreservation of NK cells is currently employed, deterioration in cell viability and functionality has been reported.^20^, ^21^, ^26^, ^27^, ^28^ This could be as a result of using DMSO, a well‐known toxic cryoprotectant.^20^, ^29^, ^30^ In addition, although such a toxic cryoprotectant is usually removed (during the unloading process) from cells after cryopreservation, the trace amounts left inside cells are associated with side‐effects in human (e.g., neurotoxicity, cardiovascular failure, respiratory arrest, and fatal arrhythmias).^31^, ^32^ The deterioration effects posed due to exposure of cells to conventional cryoprotectants can be avoided by replacing them with nontoxic bioinspired cryoprotectants. Recently, bioinspired biocompatible materials, such as ectoine and trehalose, have been utilized to cryopreserve human cells (e.g., red blood cells, oocytes, and stem cells).^33^, ^34^, ^35^, ^36^

This study describes the development of an innovative approach to cryopreserve human cytokine‐activated NK cells using a cocktail of nontoxic bioinspired cryoprotectants (i.e., dextran and carboxylated ε‐poly‐L‐lysine (CPLL)). The cells were cryopreserved using the slow‐freezing approach. The recovered cells were evaluated using a viability assay and compared to the selected control (i.e., DMSO‐based cryopreservation). Subsequently, the recovered cells were incubated with leukemia cells, and their cytotoxic functional potency was evaluated by flow cytometry.

To cryopreserve NK cells, we utilized a cocktail of biocompatible naturally occurring cryoprotective solution containing dextran and CPLL. The cocktail solution was based on experiment performed on a pool of candidates shown in Figure S1 (Supporting Information). Dextran is a bioinspired polysaccharide first discovered by Pasteur,^37^ and produced in nature by certain bacteria such as *Leuconostoc* and *Streptococcus*.^38^ The cryoprotective capability of dextran has been demonstrated for various cell types including stem cells^39^, ^40^ and red blood cells.^41^, ^42^, ^43^ PLL is a biocompatible *L*‐lysine homopolymer naturally occurring during the fermentation process of multiple bacteria such as *Streptomyces* 44, 45 (**Figure** **1**A). It has been used as a food and cell preservative^45^, ^46^, ^47^ as well as for other biomedical applications (e.g., tissue engineering and drug delivery).^48^, ^49^, ^50^ Recently, the cryoprotective property of CPLL has been reported for numerous cell types such as red blood cells and stem cells.^51^, ^52^, ^53^, ^54^ Carboxylated PLL acts in a way similar to antifreeze proteins (AFPs); however, the exact mechanism of action is still unknown.^55^, ^56^ The CPLL exhibits antifreeze activities by inhibiting ice crystal growth and recrystallization during cryopreservation.^57^, ^58^ During the cryopreservation process, small ice crystals tend to convert to large ice crystals by a process called recrystallization. This phenomenon occurs because small ice crystals have lower melting point (compared to larger crystals), and once they melt they release liquid water, which tend to merge with adjacent larger crystals and recrystallize.^55^ Therefore, ice recrystallization forms a matrix of large ice crystals, causing more damage to cells as a result of membrane rapture and cell dehydration.^55^, ^59^, ^60^ In addition, the ice crystals in CPLL are more hexagonal or bipyramid in shape since the carboxyl group binds to crystals.^51^, ^53^, ^55^ This hexagonal crystal shape reduces the damage to cell membrane, in contrast to round and flat ice crystals observed when cooling pure water.^51^, ^53^, ^61^ Furthermore, the CPLL shows high adsorption to cell membrane during cooling, which also contributes to the protection of cell membrane from outside and maintaining membrane integrity.^51^, ^62^ This might be related to the binding affinity of CPLL to cell membrane, in a similar way of AFPs.^51^ In addition, combining both dextran and CPLL might also provide synergic cryoprotective effect^62^ (Figure 1B). The high affinity of side‐chain amino groups of polyamines of CPLL and dextran molecules to cell membrane might help in maintaining the integrity of cell membrane. These molecules show high affinity to water and thus could also help removing intracellular water to the surrounding of cells during freezing. In this process, dextran and CPLL might build up at the cell boundary in such a manner that the diffusion of solutes is restricted; therefore, reducing the mechanical damage caused by ice crystal formation and growth. The combination of dextran and CPLL could also interact with concentrated salts controlling the degree of dehydration to a level sufficient to avoid intracellular ice formation during freezing. To further elucidate the exact cryoprotective mechanism(s) of the developed solution, the interactions of dextran/CPLL molecules with water, salts, and lipid membranes during freezing can be investigated using spectroscopic methods, such as solid‐state nuclear magnetic resonance.^55^, ^62^ Prior to cryopreservation, the NK cells were loaded with precooled (≈4 °C) cryoprotective cocktail solutions at different dextran (5 or 10% w/v) concentrations with CPLL (7.5% w/v) at room temperature for 5 min. A low concentration of dextran (i.e., 5%) was chosen since the results show no significant difference in cell viability between solutions containing 5% dextran compared to 10% dextran (Figure 1C). Thus, based on these data a combination of 5% dextran with 7.5% CPLL was chosen to be the basis of our subsequent experiments. In this study, we focused on replacing DMSO, a toxic cryoprotectant used for cryopreservation NK cells, with biocompatible bioinspired cryoprotectants (i.e., dextran and CPLL). In addition, our solution does not include animal or human serum, which prevents potential transmission of zoonotic infections or causing allogeneic reaction, respectively.

**Figure 1:**
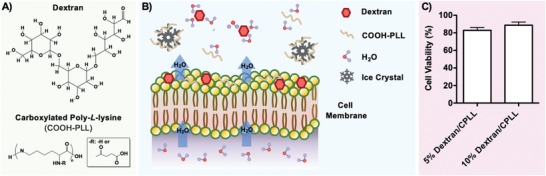
Bioinspired biocompatible cryoprotectants for cryopreservation of natural killer cells. A) Schematic showing the chemical structures of dextran and carboxylated poly‐L‐lysine (CPLL). B) Schematic demonstrating the potential mechanism of action of dextran and CPLL during cryopreservation of natural killer (NK) cells. The synergic effect of CPAs is related to their high affinity to cell membrane, water molecules, and solutes. This characteristic might provide cell protection while removing intracellular water, restricting solute diffusion, and controlling the degree of dehydration to a level sufficient to minimize intracellular ice formation during cooling. Carboxylated PLL also might limit cryoinjury to cells by binding to ice crystals and inhibiting their growth and recrystallization during rewarming. C) Determination of percentage (%) cell viability following CPA loading and unloading. Low level (i.e., 5% w/v) of dextran/CPLL‐based cocktail solution was used for subsequent experiments since there is no significant difference in cell viability between 5 and 10% dextran concentrations. The data shown are averages with standard error of the mean (SEM) from various independent experiments. For 5% dextran/CPLL group *N*_experiments_ = 3; *n*_total cells_ = 315 and for 10% dextran/CPLL *N*_experiments_ = 3; *n*_total cells_ = 416.

To achieve cryopreservation, we utilized a slow freezing method, which consists of four steps: (i) CPA loading; (ii) cooling down to cryogenic temperatures (i.e., cryopreservation); (iii) warming up to ambient temperature (i.e., rewarming or thawing); and (iv) CPA unloading (**Figure** **2**A). DMSO has shown to be toxic to cells, and the damage is proportional to the exposure time^22^, ^63^; therefore, we decided to rapidly cryopreserve cells, a process that usually takes around 5 min to load cells with CPA and transfer them to −80 °C freezer (for CPA loading step). In addition, to prevent the prolonged exposure of the cells to DMSO after thawing, we decided to immediately unload the CPA, a process that usually takes around 5 min to wash the cells by resuspending them in fresh cell media and centrifugate (for CPA unloading step). Therefore, in our protocol we decided to be consistent with loading and unloading the cells for 5 min for each step. This protocol is consistent with other studies in the literature.^25^, ^29^, ^64^ Furthermore, we decided to unload the dextran/CPLL solution from cells to be consistent with the DMSO group, and because we are not sure about their effects in circulation after transfusion, which is beyond the scope of this study. However, investigation of the systemic effect of the dextran/CPLL solution is an interesting field of further research. To evaluate the effect on cells during CPA loading and unloading, cell viability was evaluated (Figure 2B). The results demonstrated that there is no significant difference between cells loaded (for 5 min) and subsequently unloaded (for 5 min; without cryopreservation) with a dextran/CPLL‐based solution (82.8 ± 3.1%) compared to DMSO‐based solution (69 ± 16.5%) and fresh cell medium (83.3 ± 7.3%) groups. Subsequently, the NK cells were cryopreserved and stored for 1 week using a slow freezing method. Following cryopreservation and rewarming, the viability of recovered cells was evaluated (Figure 2C). The results demonstrated that there is no statistically significant difference between cells cryopreserved using dextran/CPLL‐based and DMSO‐based solutions right after thawing (73.3 ± 5.3% and 65.5 ± 1.2%, respectively). These viability results are consistent with other reports, which were based on using DMSO‐based solutions.^65^, ^66^, ^67^, ^68^, ^69^ In addition, we cultured cells after cryopreservation and rewarming for up to 1 week. Although cells in the dextran/CPLL group showed a drop in viability after 1 d in culture compared to fresh cell medium (uncryopreserved) and DMSO‐based groups, the cryopreserved cells using dextran/CPLL catch up after 1 week (Figure 2D). This observation is in agreement with other studies, which reported decline in cell viability during the first 24 h in culture.^27^, ^30^ It is important to note that the cryopreserved NK cells are intended to be used in real‐life scenarios, in which patients at clinical centers receive these cells immediately after thawing without an extended culture post‐thaw.^6^, ^7^, ^8^, ^13^, ^70^ However, their fate in circulation is an interesting field of further research.^1^, ^6^, ^8^, ^11^

**Figure 2:**
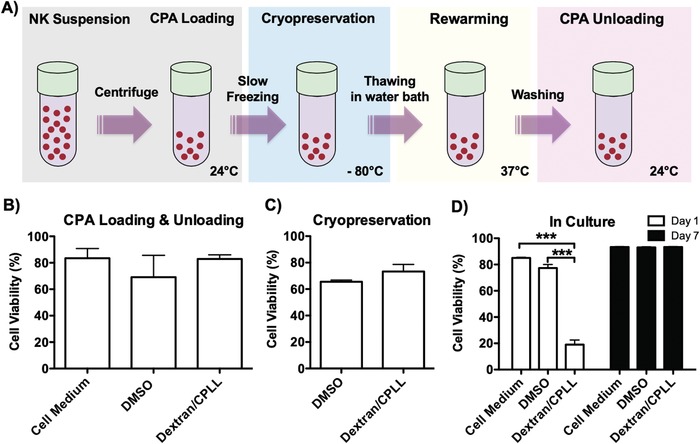
Assessment of NK cell viability following dextran/carboxylated poly‐L‐lysine (CPLL) based cryopreservation and rewarming. A) Schematic showing the cryopreservation protocol used for preservation of natural killer (NK)‐92 cells. The concentrated NK cells are loaded with bioinspired dextran/CPLL‐based cryoprotective agent (CPA) at room temperature (24 °C). The cells are subsequently placed into cryovials, cryopreserved using slow freezing method at −80 °C. The cells were then stored for 1 week. Following rapid rewarming at 37 °C, the CPAs washed out from the cells by re‐suspending the cells in NK media. B,C) Determination of percentage (%) cell viability following B) CPA loading and unloading; C) cryopreservation, rewarming, and washing the CPAs; and D) in culture for up to 1 week. The data shown are averages with standard error of the mean (SEM) from various independent experiments. For CPA loading and unloading experiments: (i) cell medium group (*N*_experiments_ = 7; *n*_total cells_ = 1316), (ii) DMSO group (*N*_experiments_ = 6; *n*_total cells_ = 877), and iii) dextran/CPLL group (*N*_experiments_ = 3; *n*_total cells_ = 315). For cryopreservation experiments: (i) DMSO group (*N*_experiments_ = 3; *n*_total cells_ = 654) and (ii) dextran/CPLL group (*N*_experiments_ = 3; *n*_total cells_ = 281). For 1 d in culture experiments: (i) cell medium group (*N*_experiments_ = 3; *n*_total cells_ = 1152), (ii) DMSO group (*N*_experiments_ = 3; *n*_total cells_ = 772), and (iii) dextran/CPLL group (*N*_experiments_ = 3; *n*_total cells_ = 416). For 1 week in culture experiments: (i) Cell medium group (*N*_experiments_ = 3; *n*_total cells_ = 3353), (ii) DMSO group (*N*_experiments_ = 3; *n*_total cells_ = 3657), and (iii) dextran/CPLL group (*N*_experiments_ = 3; *n*_total cells_ = 2219).

NK cells demonstrate cytotoxic capability to various cancer cells including leukemia cells (e.g., K562 cells).^7^, ^8^, ^71^ K562 cells are chronic myelogenous leukemia cells that have attained widespread use due to their highly sensitive in vitro target for NK cells.^72^ To evaluate cell functionality after cryopreservation and rewarming, we measured the cytotoxic potency of NK cells against K562 cells. The measurement was performed on NK cells cryopreserved with dextran/CPLL‐based and DMSO‐based solutions. Flow cytometry analysis was carried out after 4 h incubation of NK cells (effector cells) with K562 cells (target cells; prefluorescently labeled). Two different effector‐to‐target ratios (E:T: 5:1 and 10:1) were tested (**Figure** **3**A). We observed a significant (*p* < 0.05) increase of cytotoxic potency of NK cells recovered after dextran/CPLL‐based cryopreservation compared to DMSO‐based cryopreservation (Figure 3B). At both E:T ratios (5:1 and 10:1), dextran/CPLL‐based cryopreserved NK cells showed a significant killing efficiency of 67 ± 3.1% (5:1) and 71 ± 3.7% (10:1) compared to DMSO‐based cryopreserved cells 32 ± 8.2% (5:1) and 44.8 ± 3.3% (10:1). These results are in agreement with other studies, which reported a reduction of killing efficiency of NK cells cryopreserved with DMSO‐based solutions.^27^, ^28^, ^29^, ^65^, ^73^, ^74^ It is important to notice that cells cryopreserved with dextran/CPLL‐based showed higher functionality compared to cells cryopreserved with DMSO‐based solutions. This important observation indicates that there might be hidden factors in cryopreservation with dextran/CPLL‐based solution that select most potent NK cells or trigger stronger phenotypic changes of NK cells. Although, other groups have observed this phenomenon using DMSO‐based solution,^25^, ^73^ it has not been reported before using dextran/CPLL‐based solution. Therefore, such results give a hint that there is a lot of exploration needed beyond the conventional DMSO‐based solution. Although we see a higher killing efficiency of dextran/CPLL‐based cryopreserved NK cells compared to DMSO group, this was observed in a small set of experiments (*n* = 3–4). Therefore, to be able to make a strong conclusive remark on overall cell functionality, a larger sample size is required to achieve a strong statistical power analysis. Further, to evaluate the effect of dextran/CPLL‐based and DMSO‐based solutions on K652 cells, cell viability was evaluated (Figure S2, Supporting Information). The results demonstrated no significant difference in viability of K652 cells exposed to dextran/CPLL‐based solution (94.2 ± 0.6%) compared to cells exposed to a DMSO‐based solution (95.3 ± 0.3%) and a fresh cell medium (95.9 ± 0.2%). In addition, we noticed a significant difference in membrane stability of effector cells cryopreserved with both dextran/CPLL‐based and DMSO‐based solutions compared to fresh (uncryopreserved) cells (Figure S3, Supporting Information). A representative sample of a complete set of samples for each experiment was performed and its internal controls are shown in Figure S4 (Supporting Information). In addition, fresh NK and K562 cells were used as baselines to detect auto‐fluorescence or background staining (Figure S5, Supporting Information).

**Figure 3:**
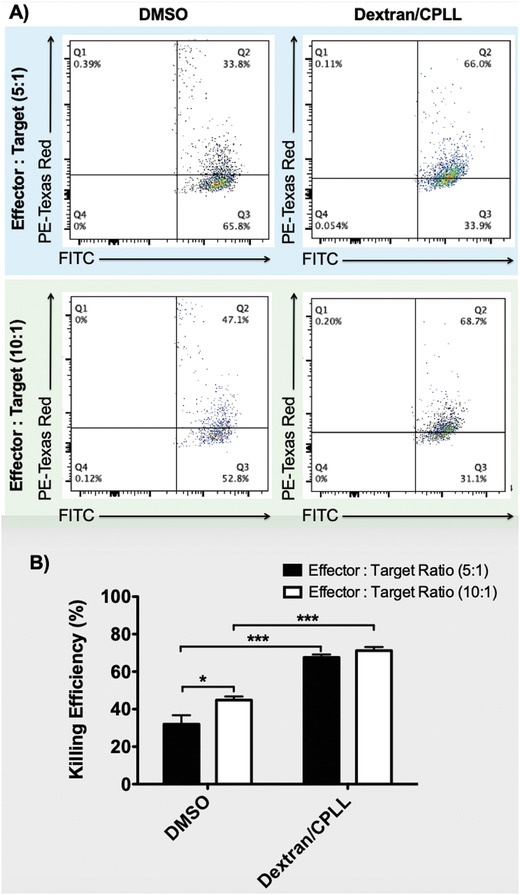
Assessment of NK cell functionality following dextran/carboxylated poly‐L‐lysine (CPLL) based cryopreservation and rewarming. Anti‐tumor functional activity of recovered NK cells after dextran/CPLL‐ and DMSO‐based solutions was evaluated against K562 leukemia cell line using cytotoxicity assay. Two different effector cells: target cells ratios were assessed (i.e., 5:1 (50 000:10 000) and 10:1 (100 000:10 000)). A) Representative flow cytometry dot plots. B) Quantification of flow cytometry analysis. The data shown are averages with standard error of the mean (SEM) from various independent experiments (*n* = 3–4).

In this study, we report the preservation of human NK cell viability and function following cryopreservation using an innovative cocktail of biocompatible bioinspired solution based on dextran and CPLL. The NK cells were cryopreserved using a slow freezing method. Right after rewarming and CPA unloading, NK cells preserved with dextran/CPLL‐based solution maintained their viability at a comparable level to DMSO, i.e., the standard cryoprotectant used in cryopreservation. However, the lower viability observed in the first day of culturing the cells cryopreserved with dextran/CPLL‐based solution indicates that this cocktail solution can be further improved. Further, we demonstrate the preservation of the anti‐tumor functional potency of recovered NK cells. These results represent an important exploration toward evaluating the phenotypic changes that occur to NK cells during cryopreservation, which might preserve their functional capabilities. The developed bioinspired cocktail solution has the potential to pave the way for the development of similar approaches, which look at the functional capability of cryopreserved NK cells using biocompatible materials available in nature with broad applications for other cell types.

## Experimental Section

*Cell Lines and Cultures—Human Cells*: Human natural killer (NK‐92) cell line and chronic myelogenous leukemia (K562) cell line used in this study were acquired from ATCC (Manassas, VA, USA).

*Cell Lines and Cultures—Cell Culture*: The NK‐92 cells were cultured in suspension in X‐VIVO (without gentamicin or phenol; Lonza, Basel, Switzerland) supplemented with human AB serum (5%; Corning, New York, USA), and the human interleukin‐2 (500 UI mL^−1^; Rehovot, Israel) was used as a cytokine for activation. The K562 cells were cultured in suspension in Iscove's Modified Dulbecco's Medium (ATCC) supplemented with fetal bovine serum (FBS; 10%; EMD Millipore, Hayward, CA) with penicillin/streptomycin (1%, Thermo Fisher Scientific, Waltham, MA, USA) and L‐glutamine (1%; Sigma‐Aldrich, St. Louis, MO, USA). All cells were maintained at concentration of 2 × 10^5^ cells mL^−1^ in 5% CO_2_ incubator at 37 °C, and the cell medium was changed every 3 d.

*Cryopreservation Procedure—Bioinspired Cryoprotective Materials*: The cryoprotective cocktail solutions were prepared using CPLL solution and various concentrations of dextran‐40 in Dulbecco's modified Eagle's medium (DMEM). ε‐poly‐L‐lysine solution (25%, Akron Biotech, West Palm Beach, FL, USA) and succinic anhydride were combined and allowed to react for 1 h at 50 °C resulting in a CPLL solution. Succinic anhydride and DMEM were both obtained from Sigma‐Aldrich (St. Louis, MO). The CPLL solution was then added to a room temperature DMEM. The CPLL and DMEM were mixed until homogenous. This medium was used as a base for the preparing solutions at various concentrations of dextran‐40 (Akron Biotech, West Palm Beach, FL, USA). Dextran‐40 used in this study has a normative molecular weight of 40 000 Da, ranging from 35 000 to 45 000 Da. The polydispersity, or *M*_w_/*M*_n_, is in the range of 1.4–1.9, as provided by the supplier. PLL has a molecular weight of 4700, with a polydispersity of 1.14. No purity is being reported for the material.

*Cryopreservation Procedure—CPA Loading*: Human NK‐92 cells were loaded with the precooled (≈4 °C) cocktail of biocompatible CPA solution (Akron Biotech, West Palm Beach, FL, USA), which consists of dextran (5% w/v (equal to 50 mg mL^−1^)) and carboxylated poly‐L‐lysine (7.5% w/v (equal to 75 mg mL^−1^)) in DMEM for 5 min at room temperature. For the control group, DMSO (10% (v/v); Sigma‐Aldrich) in FBS was loaded into cells for 5 min at room temperature. Fresh NK‐92 medium was used for the negative control group.

*Cryopreservation Procedure—Cryopreservation*: For cryopreservation, the NK‐92 cells that were loaded with different CPA (cell density: 1 million cells in 1 mL CPA solution) transferred into cryovials (Corning). The cryovials were immediately transferred into a freezing container (Mr. Frosty; Thermo Fisher Scientific) and placed into a −80 °C freezer (Thermo Fisher Scientific) for overnight. The cryovials were then transferred to a liquid nitrogen tank for storage.

*Cryopreservation Procedure—Thawing and CPA Unloading*: For thawing, the cryovials were placed into water bath (Thermo Fisher Scientific) for 1–2 min (until it was clear that the media in the cryovials were thawed). The cells were then pipetted into a 9 mL of prewarmed (≈37 °C) fresh NK‐92 cell medium, and allowed to settle for 5 min. Following that cells were centrifuged (at 1280 rpm for 5 min), media discarded, cells washed one more time, and suspended with fresh NK‐92 media.

*Viability Assay*: To evaluate the viability of NK‐92 cells after cryopreservation and rewarming, the recovered cells were stained using trypan blue (Sigma‐Aldrich), as a dye exclusion method. The live cells were then counted using a hemacytometer (Thermo Fisher Scientific). Percentage of cell viability was then calculated by dividing the number of live cells over total number (live + dead) of cells.

*Cytotoxicity Functionality Assay*: For the cytotoxicity functionality assay, the target (K562) cells were first counted and stained with calcein AM (0.1 × 10^−6^ m; BD Biosciences, San Jose, CA, USA) in phosphate‐buffered saline (PBS; Thermo Fisher Scientific) for 5 min in 5% CO_2_ incubator at 37 °C. After staining, the K562 cells were washed and suspended in Roswell Park Memorial Institute (RPMI) media (Thermo Fisher Scientific) supplemented with FBS (20%). Subsequently, the cells were plated in a 96‐well plate (Sigma‐Aldrich). Second, the activated effector (NK‐92) cells were added onto the top of target cells at different effector‐to‐target ratios (E:T: 5:1 and 10:1; Supplementary Table S2) co‐incubated for 4 h in RPMI media supplemented with FBS (20%) in the dark at 37 °C in 5% CO2 incubator. Subsequently, the dead cells in the cell mixture were stained with propidium iodide (PI; 10 µg mL^−1^; Sigma‐Aldrich) for 15 min at room temperature. The cells were then washed and suspended in flow cytometry buffer (PBS supplemented with 2% FBS). Flow cytometry assessment was immediately performed on flow cytometer (BD Biosciences, San Jose, CA, USA) with an excitation wavelength of 488 nm. The fluorescence signals were collected through a 530 nm band pass (filter for the fluorescein isothiocyanate (FITC) signals), and a 650 nm long pass filter for PI fluorescence. To determine the percent‐specific killing efficiency of NK cells, K562 cells were gated and quantified. K562 target cells that both stain for calcein‐AM and PI represented dead target cells. The effector cells, which are unstained (Q4) or stained with PI (Q1), were gated out. For 5:1 ratio, the number of gated out cells was 0.5 × 10^5^, while for 10:1 ratio, the number of gated out cells was 1 × 10^5^. Internal controls of each group and the baseline (fresh) cells were used to detect the auto‐fluorescence or background staining as well as determine the gating (Figures S4 and S5, Supporting Information). Subsequent data analysis was performed using FlowJo software (FlowJo, LLC, Ashland, OR, USA).

*Statistical Analysis*: Experiments were carried out multiple times (≥3) on different NK‐92 cultures. Means, standard deviations, and standard errors were calculated. Data were analyzed using *t*‐test and one‐way and two‐way analysis of variance (ANOVA) with Tukey's and Bonferroni's honestly significant difference (HSD) tests. Statistical significance was set at *p* < 0.05. All statistical analyses were performed with GraphPad Prism (GraphPad Software). Error bars in the figures represent the standard error of the mean (SEM).

## Conflict of Interest

Dr. U. Demirci is a founder of and has an equity interest in (i) DxNow Inc., a company that is developing microfluidic and imaging technologies; (ii) Koek Biotech, a company that is developing microfluidic IVF technologies for clinical solutions; and (iii) LEVITAS Inc., a company that develops biotechnology tools for genomic analysis in cancer. U.D.'s interests were viewed and managed in accordance with the conflict of interest policies.

## Supporting information

Supplementary
