# Supplementary material for: Bioinspired Preservation of Natural Killer Cells for Cancer Immunotherapy
Source: Adv Sci (Weinh). 2019 Jan 27;6(6):1802045. doi: 10.1002/advs.201802045 (PMC6425501; doi:10.1002/advs.201802045)
Supplement: Supplementary file 1 — Supplementary [file ADVS-6-1802045-s001.pdf]

Copyright WILEY-VCH Verlag GmbH & Co. KGaA, 69469 Weinheim, Germany, 2019.

ADVANCED SCIENCE
Open Access

Supporting Information

for *Adv. Sci.,* DOI: 10.1002/advs.201802045

Bioinspired Preservation of Natural Killer Cells for Cancer Immunotherapy

*Rami El Assal, Lotfi Abou-Elkacem, Alessandro Tocchio, Shannon Pasley, Sandro Matosevic, David L. Kaplan, Claudia Zylberberg, and Utkan Demirci**

Copyright WILEY-VCH Verlag GmbH & Co. KGaA, 69469 Weinheim, Germany, 2016.

Supporting Information

Bioinspired Preservation of Natural Killer Cells for Cancer Immunotherapy

Rami El Assal, Lotfi Abou-Elkacem, Alessandro Tocchio, Shannon Pasley, Sandro Matosevic, David L. Kaplan, Claudia Zylberberg, Utkan Demirci*

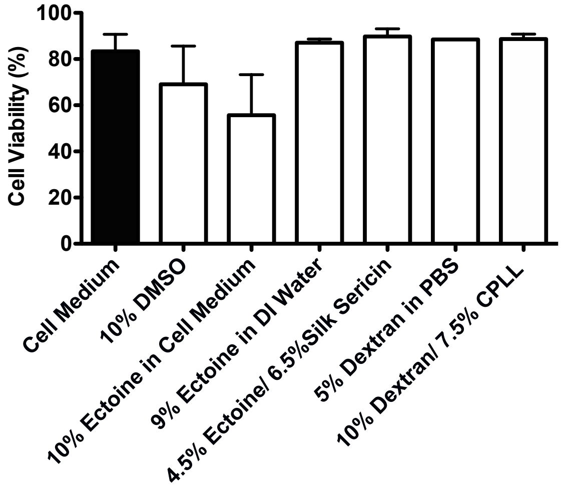

Supplementary Figure S1. Determination of percentage (%) cell viability of a pool of candidates of bioinspired cryoprotectants following CPA loading and unloading (n=1-6). Various bioinspired cryoprotectants (e.g., ectoine and silk) were tested, and dextran/ carboxylated poly-L-lysine (CPLL) cocktail solution was selected for subsequent experiments.

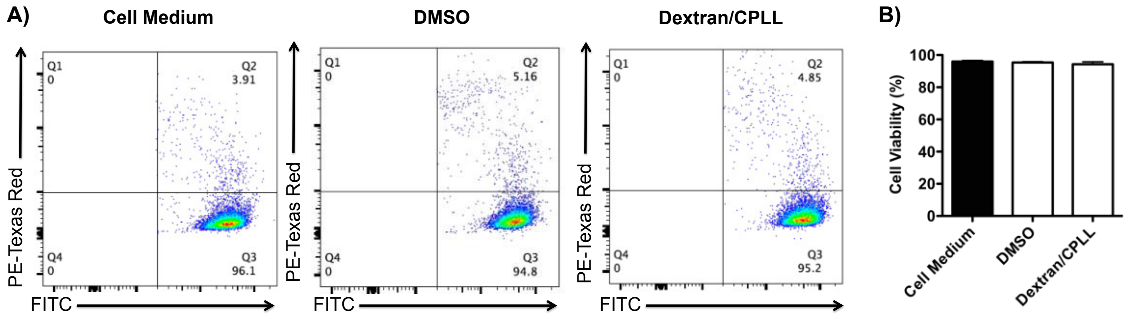

Supplementary Figure S2. Assessment of K562 cell viability following dextran/carboxylated poly-L-lysine (CPLL) loading and unloading. We performed the CPA loading for cells for 5 minutes, and the CPA unloading step was for 5 minutes in to match the NK protocol used in this study. A) Representative flow cytometry dot plots. B) Quantification of flow cytometry analysis. The data shown are averages with standard error of the mean (SEM) from various independent experiments (n=3-6).

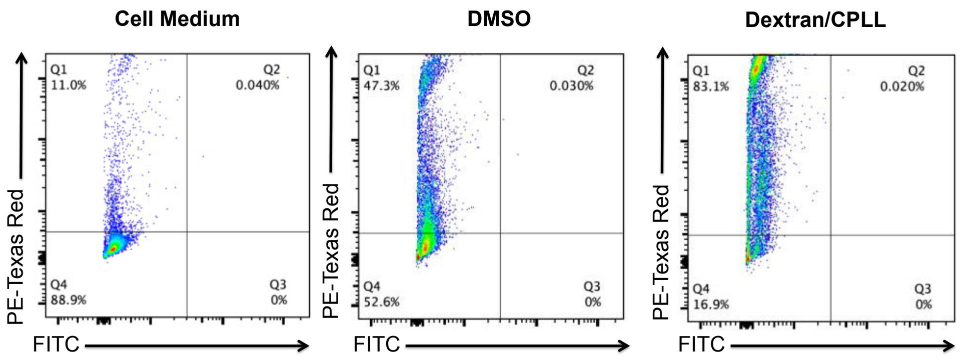

Supplementary Figure S3. Assessment of NK cell membrane stability following dextran/carboxylated poly-L-lysine (CPLL) cryopreservation and rewarming via flow cytometry.

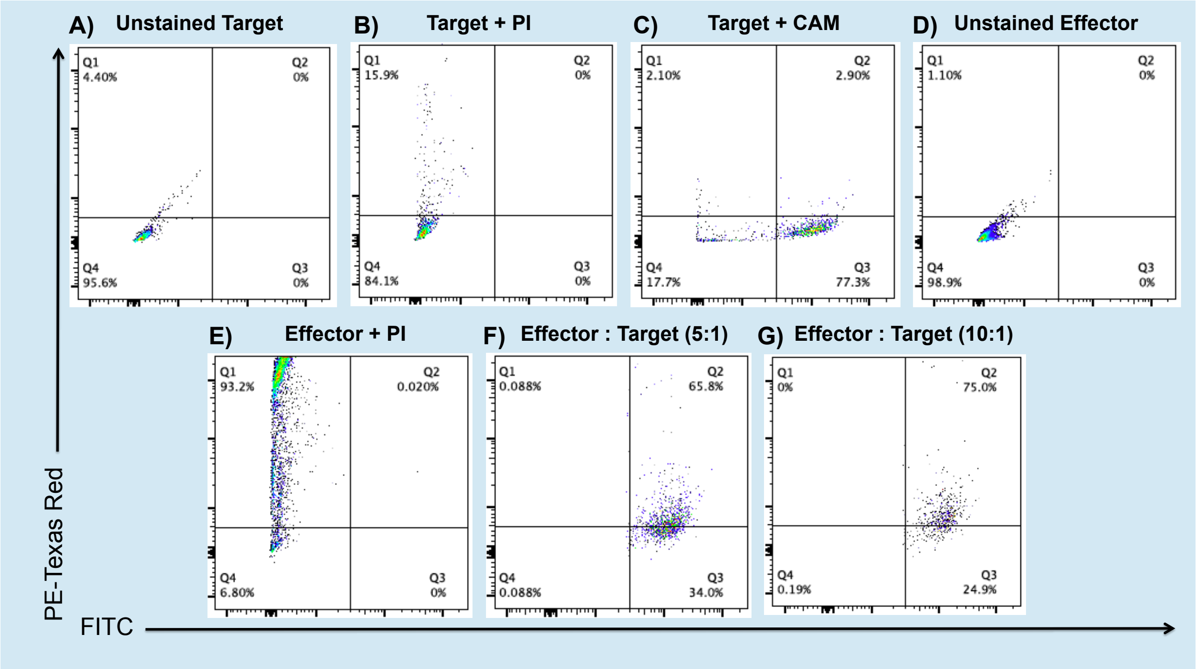

Supplementary Figure S4. A representative example of a complete set of samples for each experiment performed and it's internal controls. **A-E**) Internal controls consist of: (**A**) Unstained target cells, (**B**) target cells stained with propidium iodide (PI, as a marker of dead cells), (**C**) target cells stained with calcein AM (CAM, as a marker of viable cells), (**D**) unstained effector cells, (**E**) effector cells stained with PI. **F&G**) Main samples, which are collected and used for quantification. The data shown here are a representative of NK-cells cryopreserved with dextran/carboxylated PLL-based solution.

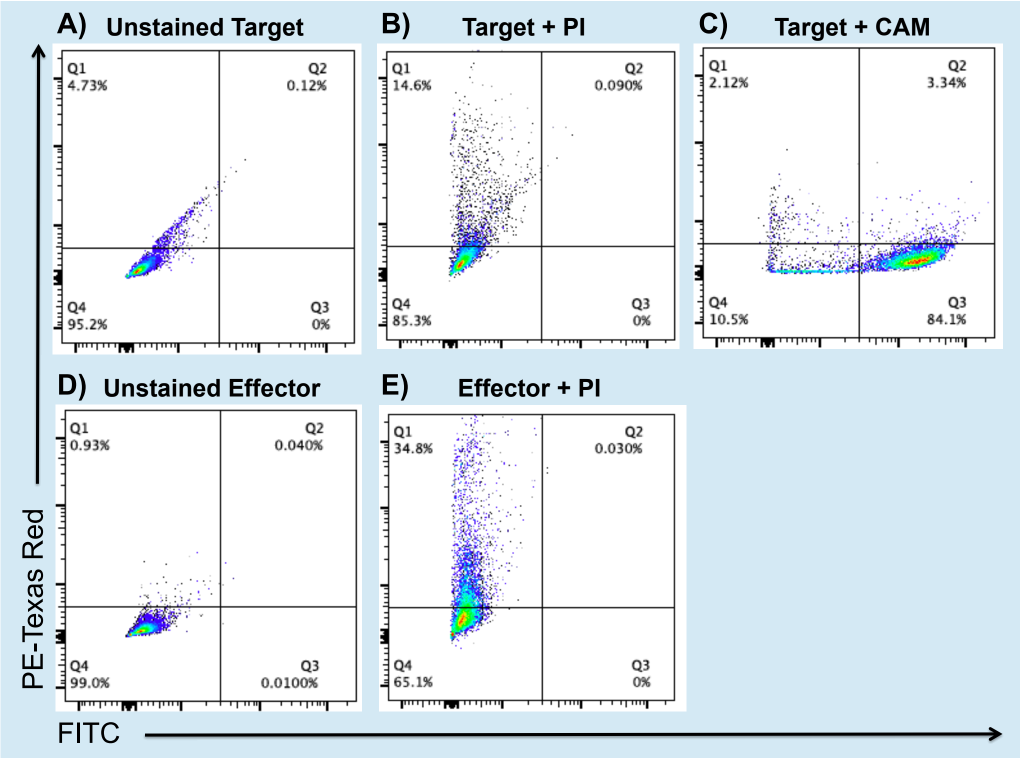

Supplementary Figure S5. Flow cytometry data of the baseline cells. Fresh NK (effector) and K562 (target) cells were used as baselines to detect the auto-fluorescence or background staining and determine the gating.

Supplementary Table S1. A summary table of existing cryoprotectants and cryopreservation methods used for NK cell cryopreservation.

#	Cell Type	CPA Type/ Concentration	Cryopreservation Method	Reference
1	NK	DMSO (10%)	Slow Freezing	Domogala, 2016
2	NK from cord blood	DMSO (10%) + Dextran 40 (5%)	Slow Freezing	Ayello, 2009
3	NK & LAK from cord blood	DMSO (10%) + Dextran 40 (5%)	Slow Freezing	Ayello, 2006
4	NK from PBMC	DMSO (10%)	Slow Freezing	Dominguez, 1997
5	NK	DMSO (20%) + FCS (30%) in RPMI (50%)	Slow Freezing	Voshol, 1993
6	LAK & NK	DMSO (10%) & 1,2-propanedio (PrOH) (10%)	Slow Freezing	Marti, 1993
7	LAK & NK	DMSO (20%) in RPMI	Slow Freezing	Letellier, 1991
8	NK	DMSO (20%) in RPMI	Slow Freezing	Kawai, 1988

Natural killer (NK) cells; Lymphocyte activated killer (LAK; add IL-2)
Peripheral blood mononuclear cells (PBMCs)

Supplementary Table S2. Cytotoxicity assay protocol.

	Sample A (T)	Sample B (T + PI*)	Sample C (T + CAM*)	Sample D (E)	Sample E (E + PI)	Sample F1 (Prestained T + E + PI)	Sample F2 (Prestained T + E + PI)
**Ratio**	-	-	-	-	-	5:1	10:1
**# Target (T) Cells**	$1x10^4$	$1x10^4$	$1x10^4$	NA	NA	$1x10^4$	$1x10^4$
**# Effector (E) Cells**	NA	NA	NA	$0.5x10^5$	$0.5x10^5$	$0.5x10^5$	$1.0x10^5$

* Propidium iodide (PI) staining.
** Calcein acetoxymethyl (CAM) ester staining.
